# Association between Serum Neopterin and Inflammatory Activation in Chronic Kidney Disease

**DOI:** 10.1155/2012/476979

**Published:** 2012-08-26

**Authors:** Ashok Kumar Yadav, Vinod Sharma, Vivekanand Jha

**Affiliations:** Departments of Nephrology and Translational and Regenerative Medicine, Postgraduate Institute of Medical Education and Research, Chandigarh 160012, India

## Abstract

*Background.* The serum levels of neopterin, a marker associated with cell-mediated immunity are elevated in chronic kidney disease (CKD). We evaluated serum neopterin levels and investigated its association with markers of inflammation in a cross-section of CKD subjects without known cardiovascular disease. *Methods.* Serum neopterin levels were measured in 118 patients with stage 3–5 CKD and 41 healthy subjects with normal kidney function (HC). Patients with known cardiovascular disease were excluded. We also estimated highly sensitive CRP (hsCRP) and interluekin-6 (IL-6), tumor necrosis factor-*α* (TNF-*α*) and interferon-*γ* (IFN-*γ*) in the CKD subjects. All assays were done using commercially available ELISA kits. The correlation between neopterin and markers of inflammation were investigated. *Results.* Of the CKD population, 82 were in stage 5 (60 stage 5 D), 24 in stage 4, and 12 in stage 3. The mean age was 51.04 ± 1.3 years and 66% were males. The commonest cause of CKD was diabetes (36%). Serum neopterin levels were 5-fold higher in CKD patients as compared to HC (74.8 ± 3.6 versus 15.0 ± 2.8 nmol/L, *P* < 0.0001). There was a graded increase of serum neopterin from stages 3 to 4 and 5. CKD 5 D patients exhibited significantly higher levels compared to nondialysis stage 5 patients (*P* < 0.0001). An inverse correlation was noted between serum neopterin and eGFR (*r* = −0.359, *P* < 0.0001). Serum neopterin correlated with hsCRP (*r* = 0.285, *P* = 0.002), IL-6 (*r* = 0.212, *P* = 0.034), and IFN-*γ* (*r* = 0.32, *P* = 0.001) but not with TNF-*α*. *Conclusion.* Serum neopterin level is elevated and correlates with the severity of CKD. The elevation correlates with elevation of most, but not all, inflammatory markers. Its role in future development of cardiovascular disease and modulation with anti-inflammatory therapies needs further studies.

## 1. Introduction 

Neopterin, a pyrazino-pyrimidine compound, is synthesized by monocytes and macrophages in response to interferon-(IFN-) *γ* produced by activated T cells. Neopterin levels are elevated in conditions of T-cell or macrophages activation [1]. It enhances macrophage cytotoxicity through its interactions with reactive oxygen, nitrogen, and chloride species [2]. 

Neopterin is a marker of cellular immune response [3]. Its levels are elevated in several conditions including autoimmune diseases such as systemic lupus erythematosus and rheumatoid arthritis [4]; infections such as hepatitis, human immunodeficiency virus, and cytomegalovirus [5–7]; cancers like hepatocellular, gastric, and urothelial carcinomas [8, 9]; congestive heart failure; coronary artery disease and myocardial infarction and transplant rejection [10–13]. Neopterin has been reported as a marker of disease progression and complication in diabetes [14].

Recent studies have shown a strong association of neopterin with CVD [15, 16]. Sasaki et al. [17] showed association of high levels with increased cardiac events rates. In a cross-sectional study, neopterin levels correlated with the extent of atherosclerosis, especially coronary and peripheral vascular disease [18]. In epidemiological studies, neopterin levels can distinguish patients with unstable angina from stable angina [12, 13]. Neopterin has been identified as a potential risk factor for cardiovascular disease in dialysis patients [19].

Chronic kidney disease (CKD) subjects are at a high risk of developing CVD. The CVD risk goes up with declining GFR and dialysis patients have a 35-fold greater CVD mortality risk [20]. High prevalence of chronic inflammation and its link to CVD, especially progressive atherosclerotic disease in CKD [21–23] have fuelled interest in understanding the extent and mechanism of this association. Elevated levels of CRP and pro-inflammatory cytokines such as interleukin- (IL-) 6 and tumor necrosis factor- (TNF-) *α* have been demonstrated to be associated with increased cardiovascular mortality in dialysis patients [24, 25]. This has led to inflammatory activation being dubbed as a nontraditional CVD risk factor in CKD. The exact understanding of how inflammation causes CVD, however, remains unclear. 

Serum and urine neopterin levels are elevated in patients with kidney diseases [26–28]. The association between inflammatory activation and neopterin has not been examined in CKD. We and others have recently shown a link between inflammation and abnormalities in T-cell subpopulation in CKD subjects [29, 30]. It is possible that these activated T-cells could mediate CVD through neopterin. We hypothesized that serum neopterin will be significantly elevated in CKD subjects and show association with other markers of inflammation.

## 2. Materials and Methods

### 2.1. Study Population

Over a 6-month period (July–December 2011), 118 patients with stage 3–5 CKD were recruited in this cross-sectional study from the outpatient clinic and ward population of nephrology department, Postgraduate Institute of Medical Education and Research, Chandigarh, a large multispecialty hospital in north India. Patients over the age of 70, those with known coronary artery disease, heart failure, malignancies, active infections and taking drugs that can modulate inflammatory response were excluded. Out of these, 58 subjects were on hemodialysis (HD) and 60 were stage 3–5 predialysis CKD subjects. Estimated glomerular filtration rate (eGFR) was calculated using MDRD formula [31], and CKD stages were defined according to KDOQI criteria [32]. Detailed history was taken and all subjects underwent a thorough clinical examination. Details of clinical features and laboratory investigations were recorded. Hypertension and diabetes were diagnosed according to the standard clinical criteria. Smoking status was categorized as current smoker and nonsmoking as never smoking and stopped smoking. As part of workup for CKD, patients were screened for evidence of CV disease, infections (including tuberculosis), and malignancies using standard tools including chest skiagram, abdominal ultrasound examination and upper GI endoscopy. All patients underwent 12-lead electrocardiogram and echocardiography. Other tests, such as stress thallium or coronary angiography were done if indicated. To evaluate the normal range of neopterin, we recruited 41 healthy volunteers from amongst the institute staff and patient's relatives with normal kidney function as judged by medical history, physical examination, urinalysis, and serum creatinine estimation. All subjects gave informed consent, and the Institution Ethics Committee approved the study. 

### 2.2. Estimation of Inflammatory Markers and Neopterin Levels

After recruitment, subjects were asked to report in a fasting state. Blood samples were collected in a vacutainer, serum was separated and stored in cryovials at −80°C until analysis. Commercially available enzyme-linked immunosorbent assays kits were used to measure serum IL-6 and TNF-*α*, IFN-*γ* (BD Biosciences, San Jones, CA), hsCRP (Diagnostics Biochem Canada Inc., London, Ontario, Canada), and neopterin (DRG International Inc, Mountainside, NJ, USA). All the samples were analyzed in duplicate. These markers were selected because of their documented association with CV complication in CKD and their association with serum neopterin level in other diseases.

### 2.3. Statistics

Data were expressed as mean ± standard error of mean (SEM) when distributed normally, or as median (interquartile range). Statistical Package for Social Sciences (SPSS) v 16.0 was used to analyze the data. Mann-Whitney **U** test, independent **t** test, and Pearson and Spearman correlation tests were performed as appropriate. All *P* values were calculated two-sided, and a value of <0.05 was considered significant.

## 3. Results

Detailed characteristics of study subjects are shown in Table 1. Most of the CKD patients were males, 85% had hypertension, 34% had diabetes, and 26% were current smokers. There was no difference in age and gender distribution between CKD and non-CKD subjects. Of the 118 CKD patients, 82 were in stage 5, 24 in stage 4, and 12 in stage 3. A total of 58 patients were on dialysis for 22.6 ± 3.0 months.

**Figure 1:**
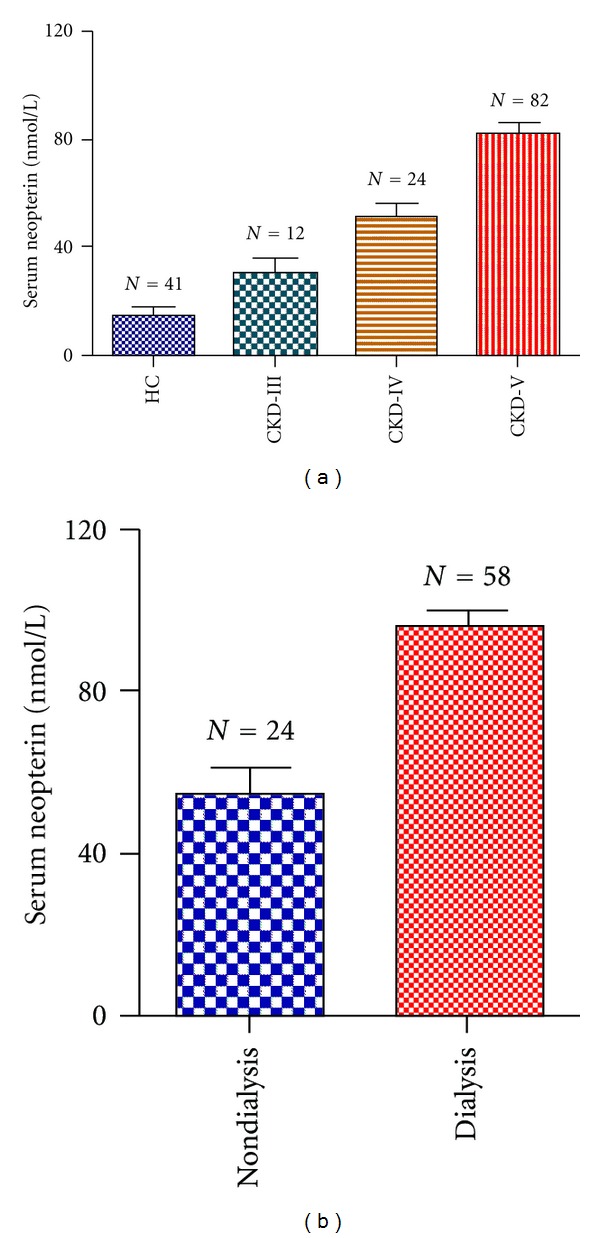
Showing (a) Serum neopterin levels in healthy subjects (HC) and in those with different stages of chronic kidney disease (CKD). **P** value are as follows: HC versus CKD III, 0.001; HC versus CKD IV, <0.0001; HC versus CKD V, 0.0001; CKD III versus CKD IV, 0.01; CKD III versus CKD V, 0.01 and CKD IV versus CKD V, 0.01, and (b) serum neopterin level in dialysis patients as compared to those nondialysis CKD patients (*P* < 0.001).

Compared to healthy subjects, serum neopterin level was approximately 5-fold higher in CKD patients (74.8 ± 3.6 versus 15.0 ± 2.8 nmol/L, *P* < 0.0001). There was no effect of diabetes, hypertension, smoking, age, or gender on the levels. A graded increase was noted in neopterin level from CKD stage 3 to 5 (29.9 ± 4.1, 51.2 ± 6, and 82.4 ± 4.2 nmol/L, *P* < 0.001, Figure 1(a)). The serum hsCRP levels were 95.7 ± 5.4 *μ*g/mL, IL-6 17.3 ± 2.6 pg/mL, TNF-*α* 7.9 ± 1.0 pg/mL, and IFN-*γ*8.3 ± 0.9 pg/mL in the CKD population.

**Figure 2:**
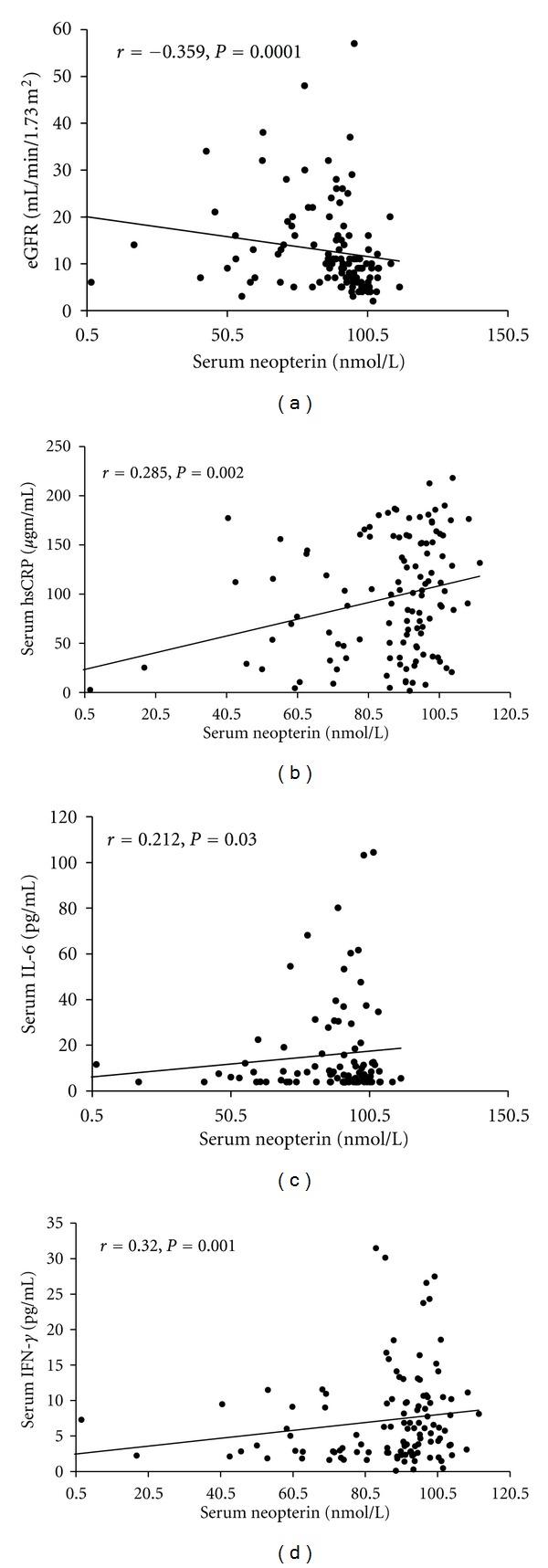
Scatter plot showing correlation of serum neopterin with (a) eGFR, (b) serum hsCRP, (c) IL-6 and (d) IFN-*γ* in CKD subjects.

The serum neopterin levels exhibited a significant inverse correlation with eGFR (*r* = −0.359, *P* < 0.0001), and had positive associations with hsCRP (*r* = 0.285, *P* = 0.002), IL-6 (*r* = 0.212, *P* = 0.03), and IFN-*γ* (*r* = 0.32, *P* = 0.001) in the CKD subjects (Figure 2). We did not find a significant correlation between neopterin and TNF-*α*.

We further subdivided stage 5 CKD patients to dialysis and nondialysis groups. Neopterin level was significantly higher in dialysis group as compared to non-dialysis group (98.8 ± 4.2 versus 58.4 ± 6.7 nmol/L, *P* < 0.0001, Figure 1(b)). There was no correlation between duration of dialysis and neopterin levels, however. The hsCRP (115.1 ± 7.3 versus 68.9 ± 11.91 *μ*g/mL, *P* = 0.002) and IFN-*γ* (10.9 ± 1.6 versus 7.0 ± 1.3 pg/mL, *P* = 0.04) were also significantly higher in dialysis group. The IL-6 level was also increased in dialysis patients but difference was not significant (19.9 ± 4.0 versus 12.1 ± 3.0 pg/mL, *P* = 0.1). No difference was noted in TNF-*α* level.

## 4. Discussion 

The present study shows that CKD patients exhibit a significant increase in serum neopterin levels compared to healthy subjects, and this increase is correlated with increased circulating levels of several markers of inflammation (hsCRP, IL-6, and IFN-*γ*). We did not note any correlation between serum neopterin and TNF-*α* levels. Neopterin levels show continuous rise with falling GFR, and are further increased in dialysis patients. The finding of inverse association of renal function with serum neopterin and a stepwise increase in serum neopterin with increasing stages of CKD is consistent with previous reports [27, 33] and suggest that impaired renal elimination and/or increased generation, or an adverse effect of inflammation on renal function might be responsible for this progressive increase.

CKD subjects showed evidence of inflammatory activation at all stages. The CRP, IL-6, IFN-*γ*, and TNF-*α* values in the CKD subjects are significantly higher than those previously reported by us in non-CKD subjects [29, 30, 34]. A heightened state of inflammation is a consistent feature of CKD, as reflected by elevated levels of IL-6 [35], IL-18 [36], leukocytes [37], fibrinogen [38], hyaluronan [39], CRP [40], and pentraxin-3 (PTX3) [41]. IL-6 is produced by activated monocytes and macrophages, activated T and B cells, and endothelial cells. In turn, IL-6 induces the release of acute phase reactants from the liver [42]. C-reactive protein is one of the major acute phase reactants, and probably acts in host defense in a manner similar to immunoglobulins [43]. Moreover, inflammatory activation has consistently been associated with CV morbidity and mortality in CKD [21–23]. Attempts to find a link between inflammation and CV disease are ongoing, but are far from conclusive. 

The positive association of serum neopterin level with hsCRP, IL-6, and IFN-*γ* in CKD suggests a link with the overall state of inflammation. Our study also demonstrated a twofold increase in neopterin levels in dialysis patients as compared to those not on dialysis, but with comparable GFR. This suggests that dialysis might further increase the production of neopterin. These subjects also showed higher levels of inflammatory markers, probably as a result of inflammatory activation and cytokine generation by activated leukocytes as a result of interaction between blood and dialysis membranes [44] which then leads to increased neopterin synthesis and/or release.

The finding of increased neopterin levels in CKD could have clinical significance. Neopterin can predict morbidity and mortality in chronic inflammatory and infectious diseases [45]. Serum neopterin was related to the anemia, weight loss, and cachexia in malignant tumors and chronic diseases (Murr G International Eurogin-east conference, Vilnius, Sept, 2001). Neopterin has been suggested as a marker of plaque progression and instability in patients with coronary artery disease [46, 47]. In a study on 2380 patients with stable angina, neopterin levels predicted future major CV events [48]. CKD is a well-known risk factor for cardiovascular disease, and it is possible that part of this increased risk could be mediated through neopterin. The adverse effects of neopterin can be due to its effect on intracellular redox state, leading to activation of constitutive and inducible NO synthase [49, 50]. Neopterin is biologically stable, and could be used as diagnostic or prognostic marker. 

Our study had some limitations. This is a cross-sectional study, and measurements have been done at a single time point. Most of our patients had newly diagnosed CKD. Whether the relationship still holds in patients on long-term dialysis is not certain. Also, whether these findings are valid for patients from other geographic areas and/or ethnic backgrounds needs to be confirmed. Our patients did not have overt CV disease but still showed rise in neopterin levels. It would be interesting to follow up these patients to see if increased levels predict future development of vascular disease. If this association is confirmed, a role could be suggested for therapies that could modulate inflammatory activation and/or target neopterin.

In summary, CKD patients exhibit elevated levels of serum monocyte activation marker neopterin. The degree of elevation correlates with the stage of kidney disease and inflammatory activation. This association needs to be confirmed in larger studies, and the link between inflammatory activation and elevation in serum neopterin levels and their contribution to the clinical events, especially CVD, requires further studies.

## Figures and Tables

**Table 1:** Characteristic of CKD study subjects.

	CKD nondialysis	CKD-Dialysis	HC
Number of cases	60	58	41
Age (yrs)	51.5 ± 1.5	52.0 ± 1.7	46.8 ± 2.2
Gender M/F	40/20	44/14	31/10
Body mass index (kg/m^2^)	22.8 ± 0.5		22.7 ± 0.3
Systolic blood pressure (mm Hg)	142.1 ± 2.3	137.9 ± 2.4	110.3 ± 2.0
Diastolic blood pressure (mm Hg)	85.2 ± 1.2	85.0 ± 1.2	80.6 ± 1.4
Diabetes	18 (30%)	22 (38%)	—
Hypertension	50 (83%)	48 (83%)	—
Current smokers	16 (27%)	15 (26%)	5 (12%)
Hemoglobin (mg/dL)	10.31 ± 0.3	9.7 ± 0.3	13.6 ± 0.6
eGFR (mL/min/1.73 m^2^)	17.0 ± 1.3	8.7 ± 1.0	106.8 ± 4.2
Total cholesterol (mg/dL)	165.9 ± 8.1	156.6 ± 6.4	
LDL cholesterol (mg/dL)	95.1 ± 8.9	88.1 ± 4.8	
Calcium (mg/dL)	7.9 ± 0.2	8.2 ± 0.2	
Inorganic phosphate (mg/dL)	6.1 ± 0.2	6.4 ± 0.3	

## References

[1] Huber C, Batchelor JR, Fuchs D (1984). Immune response-associated production of neopterin. Release from macrophages primarily under control of interferon-gamma. *Journal of Experimental Medicine*.

[2] Hoffmann G, Wirleitner B, Fuchs D (2003). Potential role of immune system activation-associated production of neopterin derivatives in humans. *Inflammation Research*.

[3] Lhee HY, Kim H, Kwan JJ, Soo SJ, Kyu BL (2006). The clinical significance of serum and urinary neopterin levels in several renal diseases. *Journal of Korean Medical Science*.

[4] Aulitzky WE, Tilg H, Nicderwieser D (1988). Comparison of serum neopterin levels and urinary neopterin excretion in renal allograft recipients. *Clinical Nephrology*.

[5] Fahey JL, Taylor JMG, Detels R (1990). The prognostic value of cellular and serologic markers in infection with human immunodeficiency virus type 1. *New England Journal of Medicine*.

[6] Halota W, Jaruga B, Pawłkowska M (2002). Serum neopterin and *β*2-microglobulin concentration as 'prognostic markers' of AIDS natural history. *Polski Merkuriusz Lekarski*.

[7] Lin CY (1989). Urinary neopterin as a new biochemical marker for the monitoring of disease activity and prognosis in membranous nephropathy associated with hepatitis B surface antigenemia. *Nephron*.

[8] Aulitzky W, Frick J, Fuchs D (1985). Significance of urinary neopterin in patients with malignant tumors of the genitourinary tract. *Cancer*.

[9] Bichler A, Fuchs D, Hausen A (1982). Urinary neopterine excretion in patients with genital cancer. *Clinical Biochemistry*.

[10] Alber HF, Duftner C, Wanitschek M (2009). Neopterin, CD4+CD28- lymphocytes and the extent and severity of coronary artery disease. *International Journal of Cardiology*.

[11] Chin GK, Adams CL, Carey BS, Shaw S, Tse WY, Kaminski ER (2008). The value of serum neopterin, interferon-gamma levels and interleukin-12B polymorphisms in predicting acute renal allograft rejection. *Clinical and Experimental Immunology*.

[12] Gupta S, Fredericks S, Schwartzman RA (1997). Serum neopterin in acute coronary syndromes. *Lancet*.

[13] Schumacher M, Halwachs G, Tatzber F (1997). Increased neopterin in patients with chronic and acute coronary syndromes. *Journal of the American College of Cardiology*.

[14] Weiss MF, Rodby RA, Justice AC (1998). Free pentosidine and neopterin as markers of progression rate in diabetic nephropathy. *Kidney International*.

[15] Auer J, Berent R, Lassnig E, Weber T, Eber B (2002). Prognostic significance of immune activation after acute coronary syndromes. *Journal of the American College of Cardiology*.

[16] Erren M, Reinecke H, Junker R (1999). Systemic inflammatory parameters in patients with atherosclerosis of the coronary and peripheral arteries. *Arteriosclerosis, Thrombosis, and Vascular Biology*.

[17] Sasaki T, Takeishi Y, Suzuki S (2010). High serum level of neopterin is a risk factor of patients with heart failure. *International Journal of Cardiology*.

[18] Hermus L, Schuitemaker JHN, Tio RA (2011). Novel serum biomarkers in carotid artery stenosis: useful to identify the vulnerable plaque?. *Clinical Biochemistry*.

[19] Avci E, Coşkun Ş, Çakir E, Kurt Y, Akgül EÖ, Bilgi C (2008). Relations between concentrations of asymmetric dimethylarginine and neopterin as potential risk factors for cardiovascular diseases in haemodialysis-treated patients. *Renal Failure*.

[20] Go AS, Chertow GM, Fan D, McCulloch CE, Hsu CY (2004). Chronic kidney disease and the risks of death, cardiovascular events, and hospitalization. *New England Journal of Medicine*.

[21] Kalantar-Zadeh K (2007). Inflammatory marker mania in chronic kidney disease: Pentraxins at the crossroad of universal soldiers of inflammation. *Clinical Journal of the American Society of Nephrology*.

[22] Stenvinkel P (2002). Inflammation in end-stage renal failure: could it be treated?. *Nephrology Dialysis Transplantation*.

[23] Stenvinkel P (2005). Inflammation in end-stage renal disease—a fire that burns within. *Contributions to Nephrology*.

[24] Bologa RM, Levine DM, Parker TS (1998). Interleukin-6 predicts hypoalbuminemia, hypocholesterolemia, and mortality in hemodialysis patients. *American Journal of Kidney Diseases*.

[25] Kimmel PL, Phillips TM, Simmens SJ (1998). Immunologic function and survival in hemodialysis patients. *Kidney International*.

[26] Fuchs D, Hausen A, Reibnegger G, Werner ER, Dittrich VP, Wachter H (1988). Neopterin levels in long-term hemodialysis. *Clinical Nephrology*.

[27] Godai K, Uemasu J, Kawasaki H (1991). Clinical significance of serum and urinary neopterins in patients with chronic renal disease. *Clinical Nephrology*.

[28] Oda K, Arai T, Nagase M (1999). Increased serum and urinary neopterin in nephrotic syndrome indicate cell-mediated immune dysfunction. *American Journal of Kidney Diseases*.

[29] Yadav AK, Jha V (2011). CD4+CD28null cells are expanded and exhibit a cytolytic profile in end-stage renal disease patients on peritoneal dialysis. *Nephrology Dialysis Transplantation*.

[30] Yadav AK, Lal A, Jha V (2011). Association of circulating fractalkine (CX3CL1) and CX3CR1(+)CD4(+) T cells with common carotid artery intima-media thickness in patients with chronic kidney disease. *Journal of Atherosclerosis and Thrombosis*.

[31] Levey AS, Bosch JP, Lewis JB, Greene T, Rogers N, Roth D (1999). A more accurate method to estimate glomerular filtration rate from serum creatinine: a new prediction equation. *Annals of Internal Medicine*.

[32] National Kidney Foundation (2002). K/DOQI clinical practice guidelines for chronic kidney disease: evaluation, classification, and stratification. *American Journal of Kidney Diseases*.

[33] Pecoits-Filho R, Heimbürger O, Bárány P (2003). Associations between circulating inflammatory markers and residual renal function in CRF patients. *American Journal of Kidney Diseases*.

[34] Jairam A, Das R, Aggarwal PK (2010). Iron status, inflammation and hepcidin in ESRD patients: the confounding role of intravenous iron therapy. *Indian Journal of Nephrology*.

[35] Pecoits-Filho R, Bárány P, Lindholm B, Heimbürger O, Stenvinkel P (2002). Interleukin-6 is an independent predictor of mortality in patients starting dialysis treatment. *Nephrology Dialysis Transplantation*.

[36] Chiang CK, Hsu SP, Pai MF (2004). Interleukin-18 is a strong predictor of hospitalization in haemodialysis patients. *Nephrology Dialysis Transplantation*.

[37] Reddan DN, Klassen PS, Szczech LA (2003). White blood cells as a novel mortality predictor in haemodialysis patients. *Nephrology Dialysis Transplantation*.

[38] Zoccali C, Mallamaci F, Tripepi G (2003). Fibrinogen, mortality and incident cardiovascular complications in end-stage renal failure. *Journal of Internal Medicine*.

[39] Stenvinkel P, Heimburger O, Wang T, Lindholm B, Bergstrom J, Elinder G (1999). High serum hyaluronan indicates poor survival in renal replacement therapy. *American Journal of Kidney Diseases*.

[40] Zimmermann J, Herrlinger S, Pruy A, Metzger T, Wanner C (1999). Inflammation enhances cardiovascular risk and mortality in hemodialysis patients. *Kidney International*.

[41] Tong M, Carrero JJ, Qureshi AR (2007). Plasma pentraxin 3 in patients with chronic kidney disease: associations with renal function, protein-energy wasting, cardiovascular disease, and mortality. *Clinical Journal of the American Society of Nephrology*.

[42] Nijsten MWN, De Groot ER, Ten Duis HJ, Klasen HJ, Hack CE, Aarden LA (1987). Serum levels of interleukin-6 and acute phase responses. *Lancet*.

[43] Schultz DR, Arnold PI (1990). Properties of four acute phase proteins: C-reactive protein, serum amyloid A protein, *α*1-acid glycoprotein, and fibrinogen. *Seminars in Arthritis and Rheumatism*.

[44] Zaoui P, Hakim RM (1994). The effects of the dialysis membrane on cytokine release. *Journal of the American Society of Nephrology*.

[45] Werner ER, Werner-Felmayer G, Fuchs D (1991). Biochemistry and function of pteridine synthesis in human and murine macrophages. *Pathobiology*.

[46] Garcia-Moll X, Coccolo F, Cole D, Kaski JC (2000). Serum Neopterin and complex stenosis morphology in patients with unstable angina. *Journal of the American College of Cardiology*.

[47] Zouridakis E, Avanzas P, Arroyo-Espliguero R, Fredericks S, Kaski JC (2004). Markers of inflammation and rapid coronary artery disease progression in patients with stable angina pectoris. *Circulation*.

[48] Pedersen ER, Midttun Ø, Ueland PM (2011). Systemic markers of interferon-*γ*-mediated immune activation and long-term prognosis in patients with stable coronary artery disease. *Arteriosclerosis, Thrombosis, and Vascular Biology*.

[49] Schobersberger W, Hoffmann G, Grote J, Wachter H, Fuchs D (1995). Induction of inducible nitric oxide synthase expression by neopterin in vascular smooth muscle cells. *FEBS Letters*.

[50] Werner-Felmayer G, Werner ER, Fuchs D (1993). Pteridine biosynthesis in human endothelial cells. Impact on nitric oxide- mediated formation of cyclic GMP. *Journal of Biological Chemistry*.

